# Transcriptome analysis reveals global regulation in response to CO_2_ supplementation in oleaginous microalga *Coccomyxa subellipsoidea* C-169

**DOI:** 10.1186/s13068-016-0571-5

**Published:** 2016-07-22

**Authors:** Huifeng Peng, Dong Wei, Gu Chen, Feng Chen

**Affiliations:** School of Food Science and Engineering, South China University of Technology, Guangzhou, 510640 People’s Republic of China; Institute for Food and Bioresource Engineering, College of Engineering, Peking University, Beijing, 100871 People’s Republic of China

**Keywords:** *Coccomyxa subellipsoidea* C-169, Elevated CO_2_, Lipid accumulation, Transcriptomic analysis, Phosphoenolpyruvate carboxylase, Pyruvate carboxylase, Carbamoyl-phosphate synthetase II, Ferredoxin, Vacuolar H^+^-ATPase, Clavaminate synthase

## Abstract

**Background:**

Microalgae are emerging as suitable feedstock for renewable biofuel production and providing a promising way to alleviate green house gas CO_2_. Characterizing the metabolic pathways involved in the biosynthesis of energy-rich compounds and their global regulation upon elevated CO_2_ is necessary to explore the mechanism underlying rapid growth and lipid accumulation, so as to realize the full potential of these organisms as energy resources.

**Results:**

In the present study, 2 and 5 % CO_2_ increased growth rate and lipid accumulation in autotrophically cultured green alga *Coccomyxa subellipsoidea* C-169. Overall biomass productivity as 222 mg L^−1^ day^−1^ and fatty acid content as 48.5 % dry cell weight were attained in 2 % CO_2_, suggesting C-169 as a great candidate for lipid production via CO_2_ supplementation. Transcriptomic analysis of 2 % against 0.04 % CO_2_-cultured C-169 unveiled the global regulation of important metabolic processes. Other than enhancing gene expression in the Calvin cycle, C-169 upregulated the expression of phosphoenolpyruvate carboxylase, pyruvate carboxylase and carbamoyl-phosphate synthetase II to enhance the anaplerotic carbon assimilation reactions upon elevated CO_2_. Upregulation of ferredoxin and ferredoxin–NADP^+^ reductase implied that plentiful energy captured through photosynthesis was transferred through ferredoxin to sustain rapid growth and lipid accumulation. Genes involved in the glycolysis, TCA cycle and oxidative phosphorylation were predominantly upregulated presumably to provide abundant intermediates and metabolic energy for anabolism. Coordinated upregulation of nitrogen acquisition and assimilation genes, together with activation of specific carbamoyl-phosphate synthetase and ornithine pathway genes, might help C-169 to maintain carbon/nitrogen balance upon elevated CO_2_. Significant downregulation of fatty acid degradation genes, as well as the upregulation of fatty acid synthesis genes at the later stage might contribute to the tremendous lipid accumulation.

**Conclusion:**

Global and collaborative regulation was employed by C-169 to assimilate more carbon and maintain carbon/nitrogen balance upon elevated CO_2_, which provide abundant carbon skeleton and affluent metabolic energy to sustain rapid growth and lipid accumulation. Data here for the first time bring significant insights into the regulatory profile of metabolism and acclimation to elevated CO_2_ in C-169, which provide important information for future metabolic engineering in the development of sustainable microalgae-based biofuels.

**Electronic supplementary material:**

The online version of this article (doi:10.1186/s13068-016-0571-5) contains supplementary material, which is available to authorized users.

## Background

Given the fact that global demand for energy resources is continuously rising while traditional fuels (e.g. fossil fuels) are non-renewable and their combustion raise numerous environmental concerns, biofuels research worldwide has been developed rapidly. Microalgae have emerged as alternative feedstock for biofuels production with several advantages such as high growth rate, high lipid yield and not competing with food crops or forestry for arable land and clean water [1, 2]. They consist of extremely diverse unicellular photosynthetic microorganisms that can fix CO_2_ and convert solar energy into chemical energy efficiently, though many issues and problems are yet to be solved for commercial feasibility [3]. On the other hand, worldwide concerns about the negative effects of climate change on human and environment have synergized the development of CO_2_ sequestration technologies, and culturing of microalgae for CO_2_ bio-fixation is one of the promising strategies [4]. The phenomenon that increased CO_2_ concentration can enhance the carbon fixation efficiency and growth rate of phytoplankton has been known for a long time [5, 6]. Additionally, studies have shown that elevated CO_2_ concentration increased lipid productivity as well as growth rate in various microalgae such as *Nannochloropsis oculata*, *Phaeodactylum tricornutum* and *Chlorella vulgaris* [7–9]. Thus, high level of CO_2_ has been applied in more than 60 species of microalgae in attempt to enhance biomass production and lipid content, as well as alleviate green house gas effects [8, 10–16]. However, most previous studies mainly focused on physiological properties of microalgae, such as growth rate, lipid content, and CO_2_ tolerance. As for mechanisms exploring, a recent study on diatom *P. tricornutum* measured the activities of seven key enzymes and their mRNA expression, and showed that pentose phosphate pathway was upregulated to maintain the NADPH supply under high CO_2_ concentrations [8]. Another recent study indicated that cAMP signaling played an important role in coordinating gene expression in diatom *Thalassiosira pseudonana* acclimation to elevated CO_2_ [17]. To our knowledge, very few researches have been reported on the global analysis of the transcripts to reveal the mechanisms underlying rapid growth and lipid accumulation upon elevated CO_2_ in microalgae.

*Coccomyxa subellipsoidea* C-169, which will be referred to as C-169 hereafter, is an elongated non-motile unicellular green alga, sizing approximately 3–9 μm. It belongs to Coccomyxa, Coccomyxaceae, Trebouxiophyceae, Chlorophyta. As the first sequenced eukaryotic microorganism from polar environment, C-169 has relatively fragile cell wall and contains more genes of enzymes involved in lipid biosynthesis and modification than any other sequenced chlorophytes [18]. Its great cold adaption capacity, together with the characteristic genome, suggested it as an attractive and promising microalga for biofuel production. However, limited effort has been made to examine the feasibility of C-169 as a prospective strain for lipid production to date. Two recent investigations showed that nitrogen starvation increased lipid content in C-169, but the biomass productivity was low, which is common in nitrogen deprivation [19, 20]. Important questions, therefore, remain. Will C-169 have higher lipid productivity and biomass under high level of CO_2_? Is there any pathway other than the Calvin cycle contributing to the assimilation of carbon upon elevated CO_2_? How can the carbon–nitrogen balance be maintained under high CO_2_ concentration? The answers to these questions are of vital importance to realize the potential of C-169 as energy resources.

In the present study, we evaluated the growth rate and lipid content in C-169 that was subjected to three different concentrations of CO_2_ (0.04, 2 and 5 %). Then, transcriptomic analysis was performed to explore the mechanism of rapid growth and lipid accumulation under CO_2_ supplementation. Our research indicated that C-169 employed global regulation to assimilate carbon and balance carbon/nitrogen metabolism to sustain rapid growth and lipid accumulation. These results provide a sharp insight into the regulatory profile upon elevated CO_2_ and a rich source of genetic information for the development of C-169 as an oleaginous microalga.

## Results

### Physiology and biochemical analysis under different CO_2_ concentrations

**Fig. 1:**
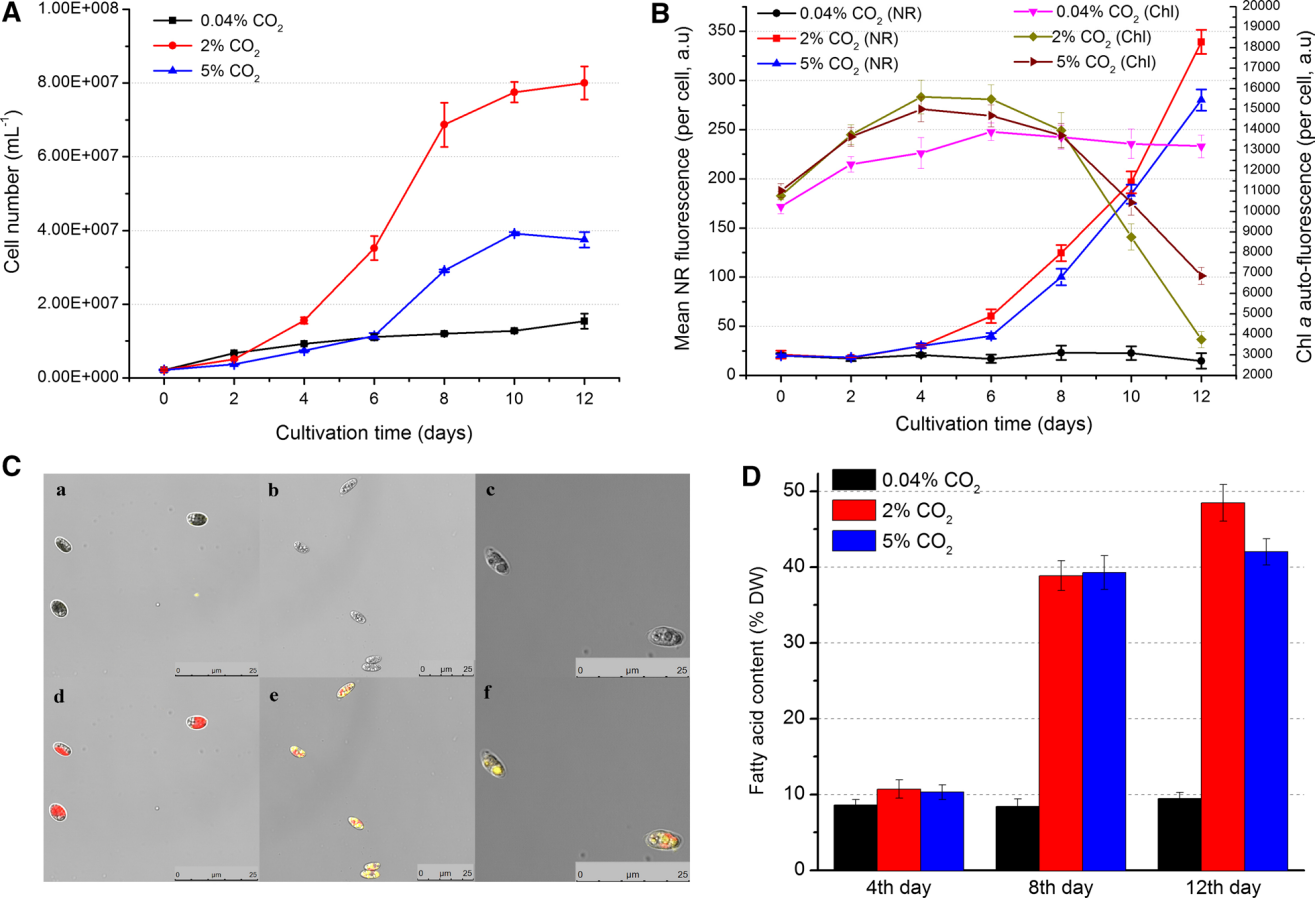
Physiological and biochemical characterization of C-169 under different CO_2_ concentrations. **A** Growth rate represented by cell numbers counted via a hemocytometer. **B** Neutral lipid and Chl *a* content represented by* fluorescence* intensity of Nile Red-stained cells and auto-fluorescence of Chl *a* throughout cultivation. All data are expressed as mean ± standard deviation (*n* = 3). **C** Microscopic images of 12-day cells captured by a confocal laser scanning microscope. *a*, *b* and *c*, respectively, represent micrograph of the algal cells with 0.04, 2 and 5 % CO_2_ under bright channel; *d*, *e* and *f* correspondingly represent those cells stained with Nile Red and viewed under fluorescence channel with *blue* light excitation. **D** Fatty acid content under different CO_2_ concentration on the 4th, 8th and 12th day

To investigate the effects of CO_2_ supplementation on the growth rate and lipid content of C-169, cells were incubated under 0.04, 2 and 5 % CO_2_. The cultivation was terminated on the 12th day when cells reached stationary phase. As shown in Fig. 1A, cell growth was markedly prompted by both 2 and 5 % CO_2_ supplementation, while 2 % CO_2_ was optimum for C-169 growth and presented a maximum cell growth rate of 0.56 day^−1^. The maximal biomass productivity for 2 % CO_2_ was 573 mg L^−1^ day^−1^, which was 627 and 88 % higher than that for 0.04 and 5 % CO_2_, respectively. The overall biomass productivity for 2 % CO_2_ was 222 mg L^−1^ day^−1^, which was 488 and 55 % higher than that for 0.04 and 5 % CO_2_, respectively. It is worth noting that 5 % CO_2_ incurred cellular aggregation in some degree (data not shown), which could partially explain the less vigorous cell growth in 5 % CO_2_ as compared to 2 % CO_2_. A remarkable increase in carbon fixation rate was observed in 2 % CO_2_ as compared to 0.04 % CO_2_, which was mainly attributed to the boosted biomass productivity rendered by 2 % CO_2_ (Table 1). The carbon fixation rate nearly doubled on the 4th day, and increased by six- to sevenfold on the 8th and 12th day.

**Table 1:** Total carbon content and carbon fixation rate

CO_2_ condition (%)	Total carbon content (% DCW)			Carbon fixation rate (g L^−1^ day^−1^)		
	4th day (%)	8th day (%)	12th day (%)	4th day	8th day	12th day
0.04	48.61 ± 0.09	48.59 ± 0.16	49.38 ± 0.30	0.107	0.075	0.068
2	50.03 ± 0.19	50.98 % ± 0.39	52.85 % ± 0.82	0.211	0.532	0.431

Flow cytometry analysis was employed to track the content of chlorophyll and neutral lipid in C-169 throughout the cultivation (Fig. 1B). On the first four days, the Chl *a* auto-fluorescence increased in all three conditions, but higher Chl a fluorescence was observed in 2 and 5 % CO_2_. It remained relatively constant in 0.04 % CO_2_ at the later stage while Chl *a* fluorescence decreased dramatically in 2 and 5 % CO_2_ after the 8th day. The decline of Chl fluorescence corresponded to the stages when the growth became stationary in 2 and 5 % CO_2_. Reduction in photosynthesis as a result of long-term elevated CO_2_ has been reported in microalgae and higher plants, for example, Arabidopsis [21]. It might be due to the light limitation and nutrient deficiency caused by rapid growth [15]. The lipid accumulation reflected by mean Nile Red (NR) fluorescence per cell remained at low level in 0.04 % CO_2_. While it was boosted dramatically in the cells cultured with 2 and 5 % CO_2_ after the 4th day and kept increasing steeply to the 12th day, the end of observation. Highest NR fluorescence was found in 2 % CO_2_. Additionally, fluorescence images of 12-day cells were taken via a confocal laser scanning microscope. Compared to cells cultured with 0.04 % CO_2_, CO_2_-supplemented cells were mainly occupied by lipid bodies (yellow), instead of chloroplasts (red) (Fig. 1C), which was consistent with the results indicated by flow cytometry analysis (Fig. 1B). These results demonstrated that CO_2_ supplementation was an effective trigger for lipid accumulation in C-169.

Fatty acid methyl esters (FAMEs) content and profile was further analyzed on the 4th, 8th, and 12th day (Fig. 1D; Table 2). Fatty acid (FA) content was comparable on the 4th day among three conditions. It remained steadily low in 0.04 % CO_2_ on the 8th and 12th day, while increased significantly in 2 and 5 % CO_2_ (Fig. 1D). The maximal FA content of 2 % CO_2_ reached 48.5 % dry cell weight (DCW) on the 12th day, which was 411.4 and 15.4 % higher than those of 0.04 and 5 % CO_2_, respectively. FA profiles indicated that C16 and C18 were the main FA components in C-169, which accounted for over 97 % of total FA (Table 2). The most remarkable change as a result of CO_2_ supplementation was observed in oleic acid (C18:1) content, whose percentage increased approximately by 6 times as compared to that of 0.04 % CO_2_ on the 12th day; while the percentage of C16:0, C18:2 and C18:3 decreased nearly by half. Such dramatic changes in FA profiles contributed to a lower degree of lipid unsaturation (DLU) with CO_2_ supplementation. The DLUs were 1.65, 1.20 and 1.19 ∇/mole for 0.04, 2 and 5 % CO_2_ on the 12th day, respectively. The favored formation of C18:1 in C-169 was also observed when cells were subjected to nitrogen deprivation [19], suggesting the similarity between the nitrogen deprivation and the later stage under CO_2_ supplementation.

**Table 2:** Fatty acid profiles under different CO_2_ concentration on the 4th, 8th and 12th day (% total fatty acid)

Fatty acids	0.04 % CO_2_			2 % CO_2_			5 % CO_2_		
	4th day	8th day	12th day	4th day	8th day	12th day	4th day	8th day	12th day
C16:0	23.92	24.69	24.00	20.94	16.63	14.54	19.28	16.63	12.54
C16:1	ND	ND	1.65	1.26	0.42	ND	1.92	0.42	ND
C16:2	6.10	7.33	7.50	4.41	1.44	0.71	4.09	2.04	0.91
C16:3	8.82	7.02	6.59	8.38	3.90	3.13	7.78	3.30	4.13
C18:0	3.21	2.47	2.11	1.00	2.22	2.16	1.42	2.02	2.16
C18:1	8.61	8.52	8.00	15.57	47.69	55.41	16.24	49.69	59.41
C18:2	23.29	24.73	26.89	21.18	13.00	11.00	21.85	11.00	10.23
C18:3	25.84	23.85	22.41	25.60	12.27	10.62	24.00	10.27	8.62
Others	0.32	1.42	0.85	1.66	2.42	2.43	3.41	4.62	2.00

*ND* not detected

### Transcriptome analysis

**Fig. 2:**
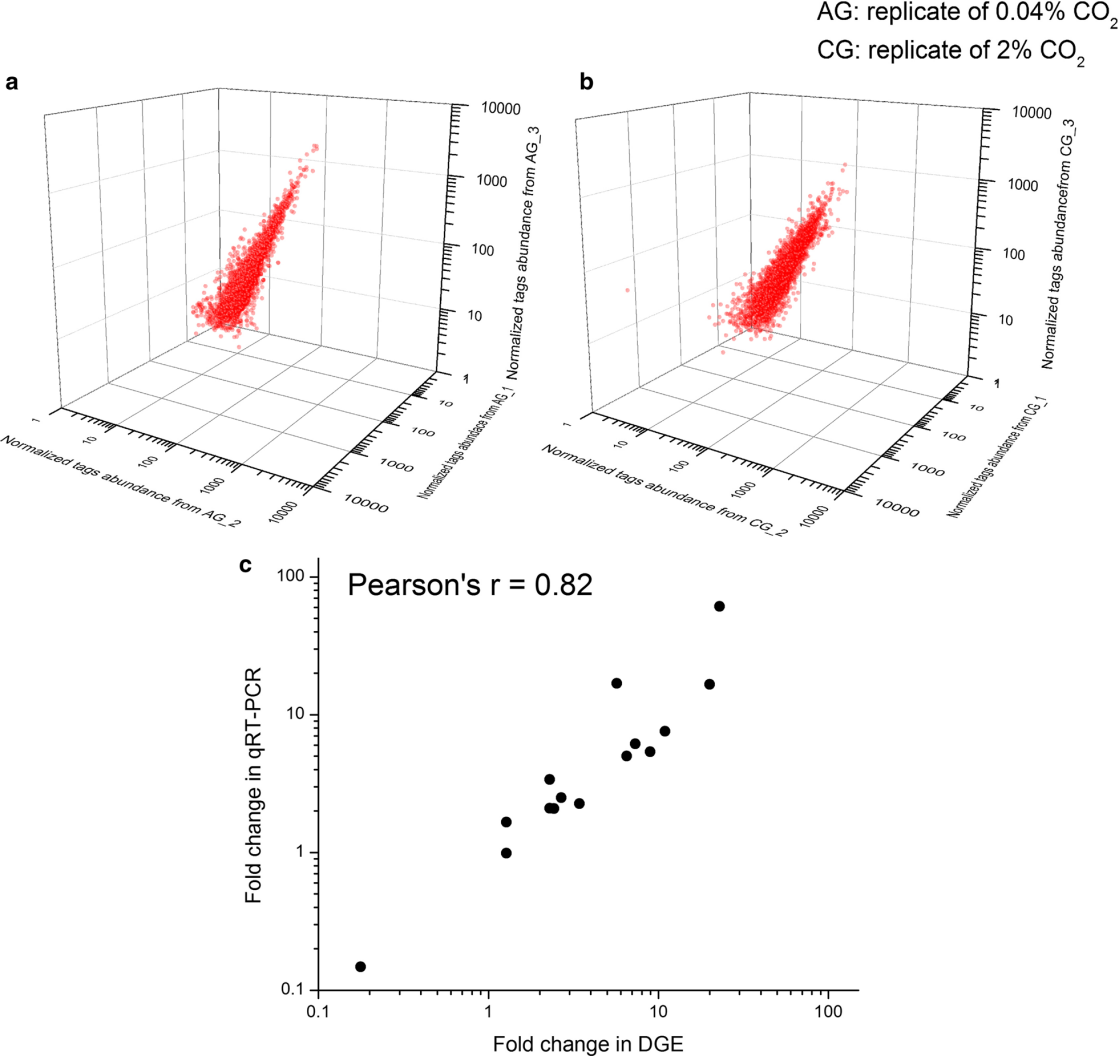
Reproducibility and reliability of the transcriptomic data. **a**, **b** The 3D scatter plots of normalized transcripts reads abundance. High correlations among three biological replicates of AG (0.04 % CO_2_) (**a**) and CG (2 % CO_2_) (**b**) indicated high degree of reproducibility of transcriptomic data. **c** DGE data were validated by quantitative RT-PCR via Pearson’s correlation coefficient

To explore the regulatory mechanisms of boosted growth and enhanced lipid content in C-169 in response to CO_2_ supplementation on transcriptomic level, cells from the 4th day were subjected to RNA extraction and digital gene expression (DGE) analysis. Three biological replicates from 0.04 % CO_2_ group (termed AG) and 2 % CO_2_ supplementation group (termed CG) were employed to guarantee statistically comparable and reliable data from DGE. Raw data ranged from 5,750,178 to 9,999,804 reads per sample. After removing the low-quality sequences and adaptor sequences, over 46 million clean reads were generated. Unambiguous reads that were uniquely matched to one gene of the reference genome with no more than one mismatch represented 9409 genes, approximately 96 % of the protein coding genes (*n* = 9851) in C-169. These reads were counted and normalized to RPKM values. Results of saturation analysis ensured that each sample had attained enough reads to approach saturation. The normalized read abundances from AG and CG libraries are compared in 3D scatter plots (Fig. 2a, b). High correlations among the three biological replicates indicated high degree of reproducibility, with an average Pearson’s correlation coefficient of *r* = 0.994 and *r* = 0.896 for the AG and CG replicates, respectively. A complete list of expression level and fold changes for all genes is presented in Additional file 1.

For brevity, unless otherwise stated, gene regulation hereafter refers to transcript abundance fold change (FC) of CG to AG. To sort out genes that were differentially regulated upon elevated CO_2_, mean RPKM values from two groups were compared. Using the criteria as |log_2_ fold change| > 1 and FDR < 0.001, 1737 differentially expressed genes (DEGs) were identified, with 871 up-regulated and 866 downregulated. To validate the DGE data, quantitative real-time PCR (qRT-PCR) analysis was performed on sixteen genes. Expression fold changes from qRT-PCR presented a high correlation with DGE (Fig. 2c), which further demonstrated the reliability of the transcriptomic data.

**Fig. 3:**
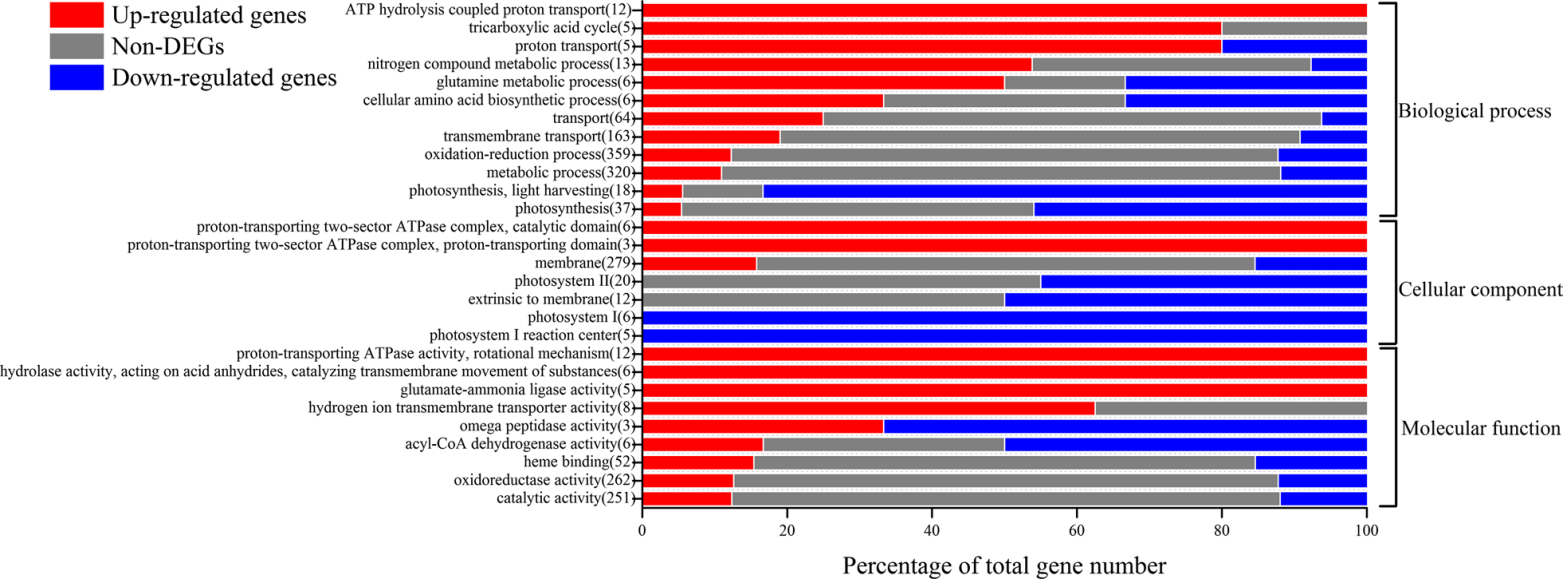
DEG-enriched GO terms. Regulatory profiles are presented as the percentage of up-regulated (*red*), downregulated genes (*blue*), and non-DEGs (*gray*) within each category of GO terms, in which DEGs were significantly enriched (*p* < 0.01)

The transcripts detected in DGE dataset were further classified based on Gene Ontology (GO) term. Totally 3901 transcripts were assigned with 1110 GO term categories according to Gene Ontology consortium [22] (Additional file 1). GO enrichment analysis of DEGs indicated that upregulated genes were enriched in ATPase, proton transporter, tricarboxylic acid (TCA) cycle, nitrogen compound metabolism, while the downregulated genes were significantly enriched in photosynthesis, including light harvesting, photosystem I and photosystem II (Fig. 3).

With the annotation information of the transcripts in C-169 available on the JGI genome portal (http://www.genome.jgi.doe.gov/pages/dynamicOrganismDownload.jsf?organism=PhytozomeV10#), an overview of metabolic pathway regulation in response to CO_2_ supplementation was generated via iPath2.0 [23] (Additional file 2: Figure S1). With the help of KEGG (http://www.kegg.jp), DEGs were assigned to 147 KEGG pathways and significantly enriched in 28 KEGG pathways with *P* value <0.05, including carbon fixation, glycolysis/gluconeogenesis, TCA cycle, pentose phosphate pathway, nitrogen metabolism, oxidative phosphorylation, etc. (Additional file 1). Exploring the variations of these pathways revealed the remodeling of metabolism in C-169 upon high CO_2_ concentration.

#### Enhanced carbon fixation in Calvin cycle

**Fig. 4:**
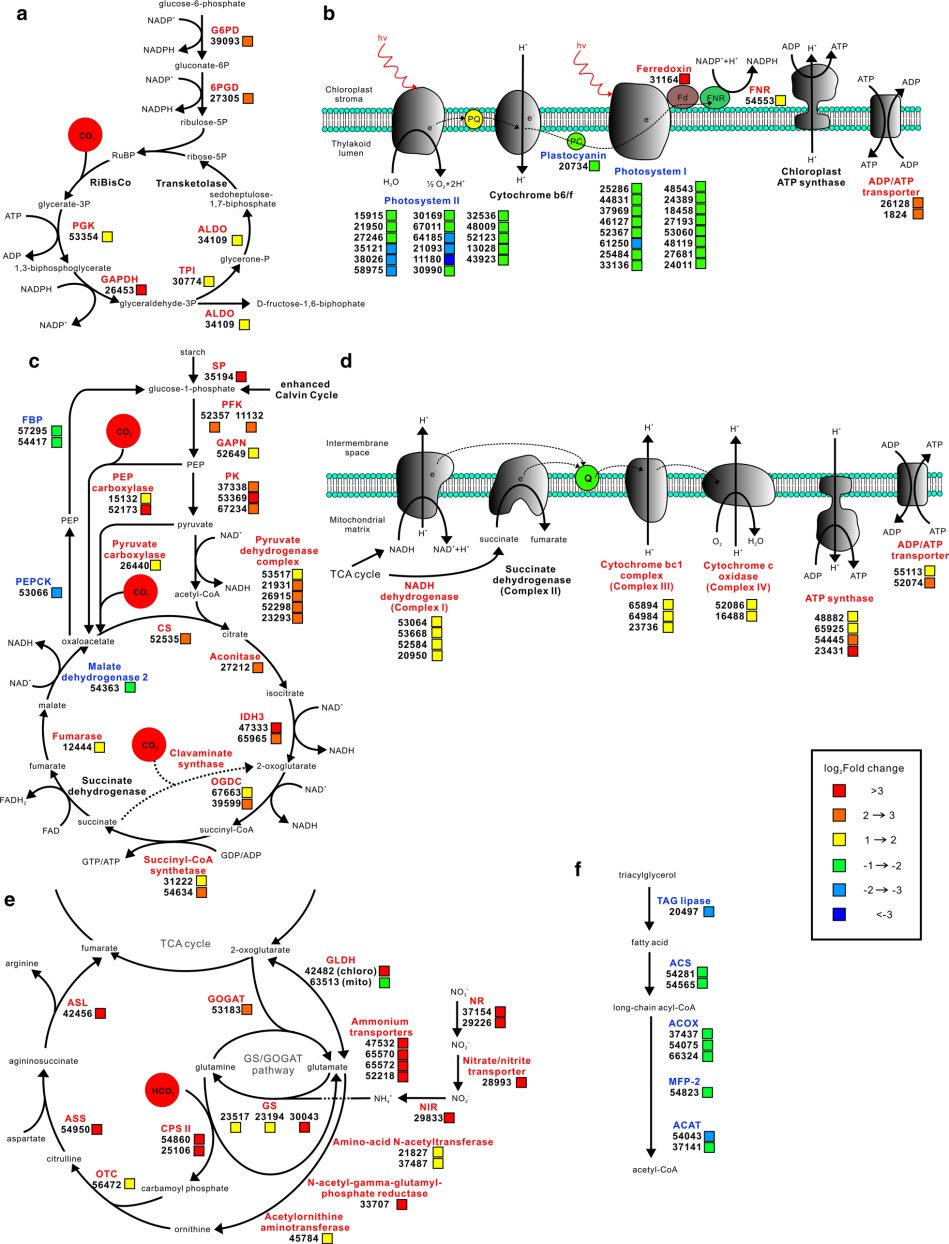
Changes in transcript abundance of genes involved in central metabolic pathways and bioprocesses in response to elevated CO_2_ in C-169. Significantly modulated pathways and bioprocesses are presented as **a** Calvin cycle and pentose phosphate pathway; **b** photosynthesis; **c** glycolysis, gluconeogenesis and TCA cycle; **d** oxidative phosphorylation; **e** nitrogen metabolism; **f** fatty acid degradation. Key enzymes are included in the map and presented as their names (in *red* up-regulated; in *blue* downregulated; in *black* relatively unchanged), gene IDs and fold changes as indicated by *color* *boxes*

Upon elevated CO_2_, genes associated with photosynthetic CO_2_ fixation, known as the Calvin cycle, were concertedly upregulated, some of which were statistically significant (Fig. 4a). Notably, genes encoding phosphoglycerate kinase (PGK) and glyceraldehyde-3-phosphate dehydrogenase (GAPDH) were significantly upregulated in CG cells. These two enzymes are crucial in the Calvin cycle, which, respectively, catalyze the phosphorylation and reduction of 3-carbon intermediates in the presence of ATP and NADPH to generate glyceraldehyde-3-phosphate. Interestingly, among four genes coding fructose-bisphosphatealdolase (ALDO) homologs, only the gene for the chloroplast homologs (34109) was upregulated to enhance conversion of glyceraldehyde-3-phosphate into d-fructose-1,6,-biphosphate. Also genes coding for enzymes in regenerating ribulose-1,5-biphosphate were upregulated coordinately. No significant upregulation was found in transcripts of RuBisCo (Ribulose-1, 5-bisphosphate carboxylase/oxygenase), the critical enzyme catalyzing the initial step of CO_2_ fixing, which might be regulated posttranscriptionally [24]. Upregulation of Calvin cycle genes implied that more carbon was fixed through the Calvin cycle to sustain rapid cell growth under high CO_2_ concentration, which was consistent with the increased carbon fixation rate in CG (Table 1). Enhanced carbon fixation raises higher demand of ATP and NADPH, which are normally generated from photophosphorylation, glycolysis, oxidative phosphorylation and pentose phosphate pathway.

#### Enhanced chloroplast oxidative pentose phosphate pathway

To fix more CO_2_ through Calvin cycle, C-169 chloroplasts required an increased supply of NADPH for the reductive reactions. Among others, oxidative pentose phosphate (OPP) pathway is a strategy to provide NADPH for biosynthetic processes. The critical enzymes for OPP pathway, glucose-6-phosphate dehydrogenase (G6PD) and 6-phosphogluconate dehydrogenase (6PGD) were found to be upregulated more than fourfold (Fig. 4a). G6PD and 6PGD participate in the irreversible reactions of OPP pathway to generate NADPH and ribulose-5-phosphate [25, 26]. Interestingly, only the chloroplast G6PD (39093) was upregulated, while the cytoplasmic G6PD (66151) did not change significantly. Thus, it was suggested that the OPP pathway in chloroplast was notably enhanced to provide NADPH for carbon assimilation, and the side product ribulose-5-phosphate was employed through the Calvin cycle.

#### Remodeling of photosynthesis

It is well established that photophosphorylation in photosynthesis provides the assimilatory power (ATP and NADPH) for the Calvin cycle. Since C-169 enhanced the Calvin cycle to fix more carbon under CO_2_ supplementation, it was assumed that the photosynthesis was also enhanced simultaneously to provide metabolic energy. However, the transcriptomic data turned out that C-169 adopted another strategy (Fig. 4b). The expression of nearly all the light harvesting center proteins, in photosystem I as Lhca and in photosystem II as Lhcb, was dramatically reduced. Correspondingly, the other components of photosystem I (PS I) and photosystem II (PS II) were notably downregulated. Though some of these components are encoded by chloroplast genome and cannot be detected by our transcriptomic analysis, expression of more than 50 % nuclear gene-encoded PSII components and all the nuclear gene-encoded PSI components were significantly suppressed. Such intensive shrink of PSI and PSII gene expression was consistent with the decreased chlorophyll fluorescence detected after the 8th day of 2 % CO_2_ (Fig. 1B). Together with the downregulation of other photosynthesis components such as the electron transporter plastocyanin, it was suggested that the photosynthesis apparatuses might be attenuated notably later. However, the expression of one of the chloroplast ferredoxins (31164) increased more than 20-fold, and ferredoxin–NADP^+^ reductase (FNR, 54553) was also upregulated. In non-cyclic photophosphorylation, ferredoxin is the last electron carrier that accepts electrons produced from sunlight-excited photosystem and transfers them to FNR to generate NADPH. Other than being the electron carriers in photosynthetic electron transport chain, ferredoxins are also electron donors of various cellular proteins, such as glutamate synthase, nitrate reductase and sulfite reductase. Notable upregulation of ferredoxin implied the active electron transport through redox state transition of ferredoxin, and suggested upregulated ferredoxin as a critical point to transfer electrons to various cellular proteins from large amount of reductant generated by enhanced photosynthesis at the early stage, which was indicated by higher Chl fluorescence during the first 6 days (Fig. 1B).

#### Enhanced glycolysis and suppressed gluconeogenesis

The gene encoding starch phosphorylase (35194) was significantly activated, indicating the upregulated starch degradation towards glucose. Coordinately, transcripts for critical enzymes in glycolysis and gluconeogenesis were found remarkably upregulated and downregulated, respectively. As the opposite catabolism and anabolism pathways of glucose, glycolysis and gluconeogenesis share most reversible enzymes while use different enzymes for the critical steps. Genes encoding critical enzymes of glycolysis, phosphofructokinase (PFK) and pyruvate kinase (PK) were found notably upregulated. These two enzymes catalyze the phosphorylation of fructose-6-phosphate to fructose-1,6-bisphosphate and the conversion of phosphoenolpyruvate (PEP) to pyruvate, respectively, which are the key regulatory steps in glycolysis. Correspondingly, genes encoding enzymes for the unique reactions in gluconeogenesis, PEP carboxykinase (PEPCK) and fructose-1,6-bisphosphatase (FBP) were significantly repressed. PEPCK catalyzes the conversion of oxaloacetate into PEP, and FBP is responsible for the conversion of fructose-1,6-biphosphate into fructose-6-phosphate. Other than cytoplasmic gluconeogenesis, FBP is also involved in chloroplast conversion of d-fructose-1,6-biphosphate into fructose-6-phosphate, using the product generated through Calvin cycle. It was interesting to find that expression of gene encoding chloroplast FBP (27479) was not significantly changed, different from its downregulated cytoplasm homologs that were involved in gluconeogenesis. Such variations indicated the precise regulation of gene expression in C-169 upon high CO_2_ concentration, and provided hints for its biotechnological modification.

All together these gene regulation data implied that CO_2_ supplementation markedly enhanced glycolysis and suppress gluconeogenesis to provide building blocks and energy for rapid growth and lipid accumulation. But it seemed that there was one exception. The gene for pyruvate carboxylase (26440), which catalyzed the first step of gluconeogenesis, was notably upregulated. However, 26440 was predicted to localize in mitochondrion by LocTREE3 [27], and the reaction product oxaloacetate actually could be fueled into TCA cycle and consumed quickly by the accelerated TCA cycle.

#### Accelerated TCA cycle and enhanced oxidative phosphorylation

In accordance with enhanced glycolysis to generate more pyruvate, more than 50 % genes encoding components of the pyruvate dehydrogenase complex were upregulated in CG (Fig. 4c). Through this multienzyme complex, pyruvate is converted into acetyl-CoA to enter the TCA cycle. Impressively, genes coding nearly all the enzymes throughout the TCA cycle were consistently upregulated, including citrate synthase, aconitase, isocitrate dehydrogenase, oxoglutarate dehydrogenase, succinyl-CoA synthetase and fumarase (Fig. 4c). Their concerted upregulation could accelerate the TCA cycle to generate more NADH and GTP/ATP. However, the last step of TCA cycle, conversion of malate to oxaloacetate might be decelerated as the gene coding mitochondria malate dehydrogenase (54363) that catalyzes the reversible conversion between malate and oxaloacetate was significantly suppressed. Such downregulation was actually coordinated with the upregulated anaplerosis of oxaloacetate by PEP carboxylase and pyruvate carboxylase. Genes for PEP carboxylase (15132, 52173) and pyruvate carboxylase (26440) were dramatically upregulated to catalyze the carboxylation of PEP and pyruvate, respectively. External CO_2_ could be incorporated through these anaplerotic reactions to generate oxaloacetate and replenish the TCA cycle. Downregulation of mitochondria malate dehydrogenase (54363) might prevent the dissipation of oxaloacetate to malate in their reversible conversion. Broadened entries of the TCA cycle suggested by upregulation in genes responsible for generating oxaloacetate and acetyl-CoA, together with the upregulation of TCA cycle genes, implied that more metabolic energy and intermediates were generated through the accelerated TCA cycle to sustain robust cell growth and anabolism in C-169 under high CO_2_ concentration.

To further investigate whether the carboxylase activities were increased in CG cells as suggested by transcriptomic analysis, the activities of PEP carboxylase (PEPcase) and pyruvate carboxylase on the 4th day were analyzed. As expected, CG cells exhibited higher PEPcase and pyruvate carboxylase activities, which were 78.6 and 46.2 % higher than those of AG cells, respectively. Thus, it was inferred that the upregulated PEPcase and pyruvate carboxylase intensified the incorporation of external CO_2_, which led to the higher carbon fixation rate in CG (Table 1).

Metabolic energy and electrons captured in the form of reduced coenzymes, NADH or FADH2, are passed through electron transport chain to generate ATP in oxidative phosphorylation apparatus within the inner membrane of mitochondria (Fig. 4d). In accordance with accelerated TCA cycle, genes involved in electron transport and oxidative phosphorylation were found remarkably upregulated, including several subunits of the Complex I (NADH dehydrogenase), III (cytochrome bc1 complex), IV (cytochrome c oxidase) and ATP synthase. Through the upregulated NADH dehydrogenase and cytochrome bc1 complex, more electrons carried by NADH and FADH2 might be passed to upregulated cytochrome C oxidase, and finally reached O_2_, the terminal electron acceptor. Using the proton gradients across the inner mitochondrial membrane generated by electron transport, ATP is synthesized by ATP synthase. Dramatically, more than 50 % genes coding ATP synthase were upregulated in CG (Fig. 4d). The metabolic energy from accelerated oxidation of nutrients and intermediates might finally be utilized to synthesize more ATP. Correspondingly, ADP/ATP transporter, the integral membrane protein responsible for transporting ATP outside and ADP inside mitochondria, was significantly upregulated. Therefore, it was inferred that the accelerated TCA cycle and enhanced oxidative phosphorylation ensured the adequate supply of ATP for sustaining rapid growth and biosynthetic processes under high CO_2_ concentration.

#### Upregulated nitrogen acquisition and assimilation

Since the carbon/nitrogen (C/N) balance is critical for cell growth, genes involved in nitrogen acquisition and assimilation showed strong upregulation in CG (Fig. 4e). The genes coding nitrate/nitrite transporter and several ammonium transporters were all dramatically upregulated, ranging from nearly 10- to more than 100-fold. Both nitrate reductase and nitrite reductase were notably upregulated to assimilate extracellular nitrogen into ammonium.

Among the three enzymatic reactions introducing ammonium into organic molecules, glutamate dehydrogenase (GLDH) and glutamine synthetase (GS) are responsible for most of the ammonium assimilated into carbon compounds under nitrogen-rich environment; while the third one, carbamoyl-phosphate synthetase (CPS), was important during nitrogen starvation [24, 28, 29]. In CG, the gene coding mitochondrion GLDH was downregulated, while the chloroplast GLDH was upregulated. Other than synthesizing glutamate, GLDH also acts in the catabolic direction to generate 2-oxoglutarate from glutamate. Given the highly suppressed gene expression of photosynthetic apparatuses and the decrease in chlorophyll fluorescence at the later stage in CG, upregulation of chloroplast GLDH might play a prominent role in the catabolism and cannibalization of photosynthetic proteins at the later stage. Three out of four glutamine synthetase (GS) homologs were upregulated significantly. GS catalyzes the ATP-dependent amidation of glutamate to form glutamine. As the most abundant amino acid in many organisms, glutamine is a major nitrogen donor in the biosynthesis of many organic N compounds such as purines, pyrimidines, and other amino acids. Thus, the upregulated GS might supply substantial nitrogen for cellular anabolism. Other than GLDH, there was an alternative mode to replenish the glutamate consumed by the upregulated GS reaction. The gene for glutamate synthase (also known as GOGAT, glutamate oxoglutarate aminotransferase) was notably upregulated. Upregulation of GS/GOGAT pathway was also observed in diatom *P. tricornutum* under nitrogen stress [28].

Instead of having carbamoyl-phosphate synthetase I (CPS I) that catalyzes the incorporation of ammonium with bicarbonate to generate carbamoyl-phosphate, C-169 contains CPS II that synthesizes carbamoyl-phosphate with glutamine as amido-N-donor (Fig. 4e). Both the large and small subunits (25106, 54860) of CPS II were significantly upregulated. Together, upregulated GS/GOGAT and CPSII constituted an enhanced ammonium assimilation pathway to incorporate bicarbonate into carbamoyl-phosphate, which is the precursor for arginine and pyrimidine synthesis and intermediate in the ornithine–urea cycle. Genes involved in the next several reactions of ornithine pathway showed consistent upregulation to generate arginine and fumarate, including ornithine transcarboxylase (OTC), argininosuccinate synthase (ASS) and argininosuccinate lyase (ASL) (Fig. 4e). C-169 does not possess a complete urea cycle, since it lacks the gene for arginase, the last enzyme of urea cycle to breakdown arginine into urea and regenerate ornithine [29]. However, genes involved in the alternative route to generate ornithine from glutamate showed strong upregulation. Thus, C-169 might employ GS/GOGAT and the specific ornithine pathway to incorporate ammonium and bicarbonate into arginine and replenish the TCA cycle through fumarate upon elevated CO_2_. The ammonium and bicarbonate might come from extracellular environment, given the high CO_2_ concentration and intensified nitrogen acquisition and assimilation; they might also be derived from the catabolism and cannibalization of preexistent amino acid and protein.

#### Lipid metabolism

Suppressed triacylglycerol (TAG) hydrolysis and fatty acid (FA) degradation was found in CG cells (Fig. 4f). The expression of TAG lipase (20497) that catalyzed the hydrolysis of TAG was significantly repressed. Genes involved in FA β-oxidation were generally downregulated, some of which reached significant level. Acyl-CoA synthetase (ACS) catalyzes the initial step of FA degradation through activation of FA with Coenzyme A. Two out of five ACS homologs were significantly downregulated. Following FA activation, cyclic reactions lead to a complete degradation of FA via the repeated cleavage of acetate units from the thiol end of FA [30]. Genes involved in these repetitive reactions showed consistent downregulation in CG cells, including genes coding acyl-CoA oxidase (ACOX), enoyl-CoA hydratase/3-hydroxyacyl-CoA dehydrogenase (MFP-2) and acetyl-CoA acyltransferase (ACAT). Lipid hydrolysis and FA degradation yield large amount of ATP through complete oxidation. Given the accelerated TCA cycle and enhanced oxidative phosphorylation in CG cells, it seemed that enough metabolic energy could be generated through these routes. Thus, lipid hydrolysis and FA degradation might be slowed down, which contributed to the increased lipid content observed at the later stage of CG cells.

Though the lipid content was slightly higher in CG than AG cells on the 4th day, it was subsequently enhanced over time in CG cells (Fig. 1D). Genes involved in lipid biosynthesis were usually reported to be upregulated in lipid-producing microalgae [19]. But on the 4th day, when the FA content difference was relatively small between 0.04 and 2 % CO_2_, it was reasonable to observe that the upregulation of genes involved in fatty acid biosynthesis and elongation was not as remarkable as the downregulation of genes involved in fatty acid degradation. Using the strict criteria of DEG as |log_2_ fold change| > 1 and FDR < 0.001, only one gene involved in fatty acid biosynthesis and elongation, 48328 (fatty acid elongase 3-ketoacyl-CoA synthase 1) was significantly upregulated on the 4th day. However, a series of genes were found upregulated when using the less strict criteria as |log_2_ fold change| > 1 and FDR < 0.05 (Additional file 1). They included genes encoding acetyl-CoA carboxylase subunit (ACCase subunit, 65159), fatty acid synthase (FAS, 49000) and several 3-ketoacyl-CoA synthases. ACCase catalyzes the first and committed step of FA biosynthesis through generation of malonyl-CoA from acetyl-CoA [31]. Subsequently, malonyl-CoA is transferred to an acyl-carrier protein, which is followed by a series of repetitive reactions catalyzed by FAS. Based on annotation of genomic data, C-169 is the only known Plantae member to have both the plant-type FAS (FAS II) and the animal-type FAS (FAS I) [18]. In CG cells, gene coding one FAS I (49000) was upregulated, thus providing experimental evidence that the animal-type FAS was functional in C-169 and contributed to lipid accumulation upon elevated CO_2_. Five 3-ketoacyl-CoA synthase homologs, 48328, 12119, 12451, 18441 and 64433, were also upregulated to build the long-chain fatty acid. Increased gene expression of ACCase, FAS and 3-ketoacyl-CoA synthases implied an upregulated trend in fatty acid biosynthesis, which should be investigated further at the later stage of elevated CO_2_.

#### Other DEG-enriched gene families

Last but not least, DEGs were interestingly found to enrich in some gene families other than those mentioned above. Among the 100 most upregulated genes, 15 encode transporters for different nutrients (Additional file 1). They were, for example, urea transporter (53548, 30678), sodium/sulfate symporter (54015), nucleoside transporter (62444), amino acid transporter (36205), sodium/dicarboxylate symporter (17172), sodium-dependent phosphate transporter (13678), as well as the ammonium transporter (65570, 65572, 65518). Thus, on the 4th day, CG cells mobilized many transporters for various nutrients to sustain rapid growth.

Active transportation was also found inside CG cells. Eight out of twenty-one kinesin family members were significantly upregulated in CG cells (Additional file 1). Four of them (20645, 13773, 37341, 15452) were among the 100 most upregulated DEGs. Kinesins are motor proteins that walk towards the positive end of microtubules [32, 33]. They transport protein and membrane components from the center of the cell towards the periphery. Such consistent and strong upregulation of kinesin family members indicated energetic metabolism inside CG cells to sustain rapid growth and lipid accumulation at the later stage.

It is noteworthy that about dozen genes encoding subunits of vacuolar H^+^-ATPase (V-ATPase) were significantly upregulated, for example, 52641, 8240, 28885 and 32039 (Additional file 1). V-ATPases couple the energy of ATP hydrolysis to transport proton across plasma and intracellular membranes. In other characterized organisms, V-ATPases are found within the membranes of many organelles, such as endosomes, lysosomes, and secretory vesicles, where they are involved in processes such as pH homeostasis and coupled transportation [34]. Given that high CO_2_ supplementation results in acidification of medium because additional carbonic acid is generated due to the solubilization of CO_2_ into aqueous phase, it might reduce the intracellular pH of CG cells and become an acid stress to C-169. Upregulation of V-ATPase might be one of the strategies to acclimate to acidification rendered by high CO_2_.

### Quantitative RT-PCR analysis at the later stage

**Fig. 5:**
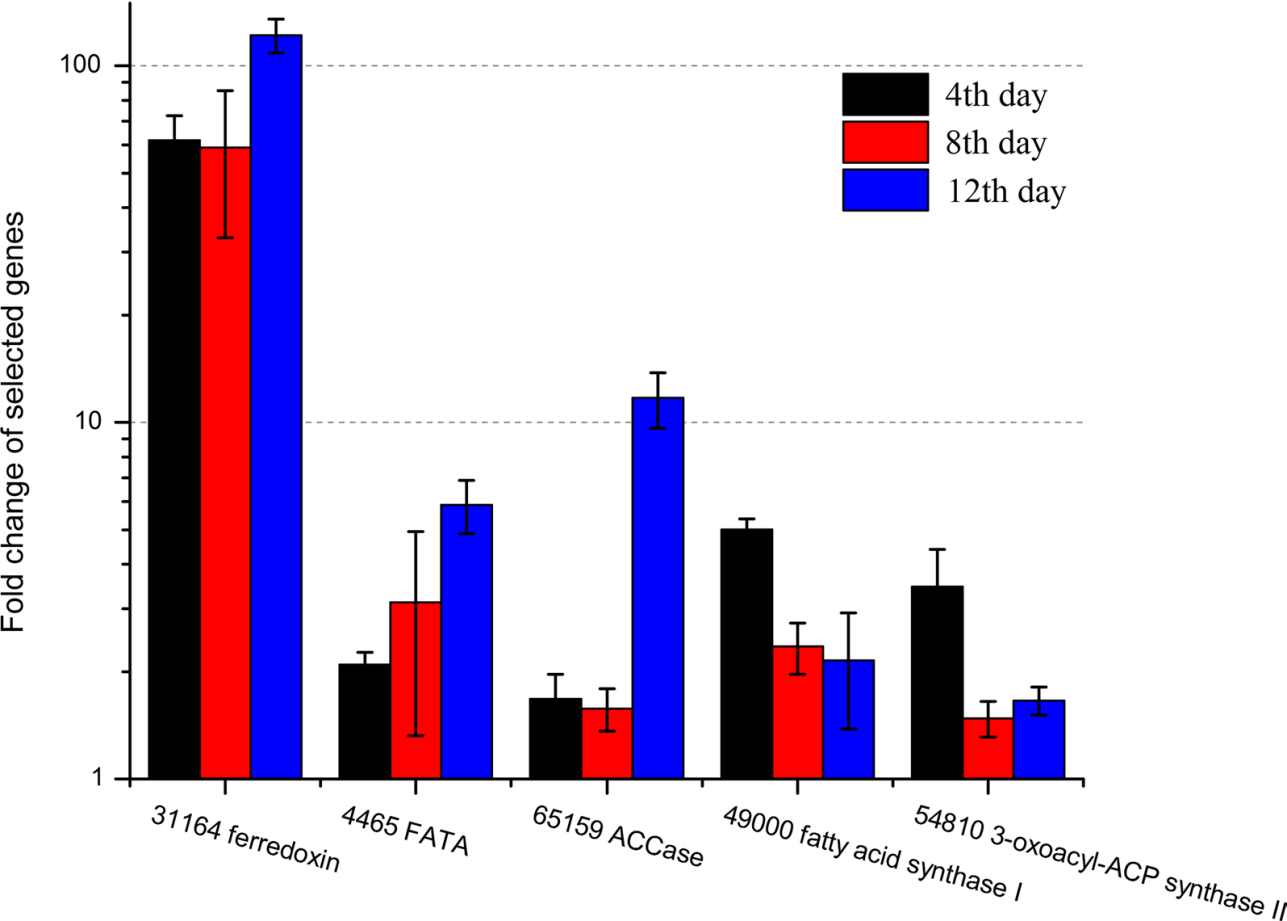
Quantitative RT-PCR analyses at the later stage. Expression fold change (CG/AG) of five genes, ferredoxin (31164) and four fatty acid synthesis genes, coding acyl-ACP thioesterase (4465), ACCase (65159), FAS I (49000), and 3-oxoacyl-ACP synthase II (54810), were evaluated on the 4th, 8th and 12th day by quantitative RT-PCR

To further reveal the gene expression at the later stage, RNAs were extracted from AG and CG cells harvested on the 4th, 8th and 12th day and analyzed by quantitative RT-PCR (Fig. 5). Four fatty acid synthesis genes, coding ACCase (65159), acyl-ACP thioesterase (4465), FAS I (49000), and 3-oxoacyl-ACP synthase II (54810), were all significantly upregulated on the 4th, 8th and 12th day, which might explain the increasing lipid accumulation in the CG cells. Compared with 49000 and 54810, stronger upregulation was observed in 4465 and 65159; their transcripts increased by about 6- and 12-fold on the 12th day upon elevated CO_2_, respectively, which suggested their tremendous contribution to lipid accumulation at the later stage (Fig. 1D). It was also intriguing to find that ferredoxin gene (31164) was activated overtime and increased by 122-fold on the 12th day. Such dramatic upregulation was consistent with the increasing trend of lipid content that required large amount of reducing equivalents and provide hints for further biotechnological application. But the detailed relationship between ferredoxin and lipid accumulation awaits further investigation.

## Discussion

Previous studies showed that extensive experiments have been conducted to prompt microalgae-derived lipid production via nutrient deficiency, especially using nitrogen starvation [35–37]. However, the disadvantage is that microalgal growth may be compromised in some degree under nutrient deficiency [19]. CO_2_ supplementation might overcome this disadvantage to some extent. Here in this study, the overall biomass productivity is 222 mg L^−1^ day^−1^ and the maximal fatty acid content is 48.5 % dry cell weight in 2 % CO_2_. They were higher than a recent report that TAG in C-169 linearly accumulated to 12.8 % dry weight after 10 day of nitrogen starvation [20], thus confirmed the great potential of lipid production from C-169 via CO_2_ supplementation. Transcriptomic analysis on the 4th day between 2 and 0.04 % CO_2_, for the first time, provides a comprehensive overview on the global regulation of important metabolic processes upon elevated CO_2_.

Photosynthetic carbon fixation has been well known as the main carbon assimilation pathway and was the focus of research on microalgae subjected to elevated CO_2_ [8]. However, our transcriptomic data here indicated that other than the enhanced Calvin cycle, C-169 mobilized several other carbon assimilation strategies to incorporate abundant carbon, and some of which were even confirmed by the enzymatic activity assay. Gene expression and enzyme activities of PEP carboxylase and pyruvate carboxylase were significantly upregulated to integrate CO_2_ to form oxaloacetate, which was an important metabolic intermediate and replenish the TCA cycle directly. Carbon was also assimilated with nitrogen into carbamoyl-phosphate by CPS II, whose transcripts increased by more than eightfold. Among the forty most upregulated genes, those encoding urea carboxylase (19857) and four clavaminate synthase-like proteins (52967, 33873, 33874, 54671) conspicuously showed up. Their expression rose 30- to more than 100-fold from very low basal level, suggesting that they were specifically activated by elevated CO_2_. Clavaminate synthase catalyzes the reversible conversion between 2-oxoglutarate and succinate [38, 39]. Given the high CO_2_ concentration, it was more likely that the reaction proceeded in the direction of succinate carboxylation, rather than 2-oxoglutarate oxidation; thus it might provide another carbon assimilation pathway to reinforce TCA cycle directly (Fig. 4c). Further experiments are needed to verify this speculation. Urea carboxylase forms carbon–nitrogen bonds between urea and bicarbonate to generate urea-1-carboxylate [40, 41]. Its upregulation implied the active cannibalization of old proteins to synthesize new intermediates.

Interesting remodeling of photosynthesis was revealed in C-169 in response to elevated CO_2_. Though Chl fluorescence does not represent the photosynthetic capacity directly, its fluctuation is positively correlated with photosystem activity. Chl fluorescence was enhanced by CO_2_ supplementation during the first 4 days while it was dramatically reduced after the 8th day (Fig. 1B). This phenomenon is consistent with previous report on plants that photosynthesis and growth rate were enhanced by short-term, but decreased by long-term elevated CO_2_ [21, 42]. Downregulation of photosystem at the later stage was implied by the notable decrease in most components of PS I, PS II and plastocyanin on the 4th day (Fig. 4b). Intriguingly, genes for the final part of photosynthetic electron transfer, ferredoxin and FNR, as well as the ADP/ATP transporter were dramatically upregulated. It seemed that plenty of light energy captured by the enhanced photosystem during the early stage was somehow converted and transported as reductant potential through ferredoxin. The quantitative RT-PCR analysis on the 4th, 8th and 12th day cells showed that ferredoxin 31164 was continuously upregulated, which might collaborate with upregulated FNR (54553) and ferredoxin–nitrite reductase (29833) to sustain anabolism, especially the lipid accumulation at the later stage. Previous investigation on diatom pointed out that nitrogen deficiency led to repression of photosynthetic protein including FNR [43]. Thus, here the uncoordinated regulation in photosystems, ferredoxin and FNR might be a special mechanism to sustain rapid growth and lipid accumulation upon elevated CO_2_, which was different from nitrogen deficiency. Except the energy transferred by ferredoxin, plenty of carbohydrates synthesized during the early stage upon elevated CO_2_ might go through the significantly enhanced glycolysis, accelerated TCA cycle and activated oxidative phosphorylation to generate large amount of intermediates, ATP and NADH to maintain rapid growth and lipid accumulation. The active intracellular metabolism was also indicated by the dramatically upregulated kinesin family members, which were responsible for intracellular transportation.

Nearly 50-fold elevated CO_2_ obviously would disrupt the C/N balance in C-169, and rapid growth resulted in greater consumption of other nutrients, especially nitrogen. Therefore, long-term elevated CO_2_ might mimic the nitrogen depletion. Actually, genes involved in nitrogen acquisition and assimilation were concertedly upregulated (Fig. 4e), which was similar with the metabolism remodeling in the diatom *P. tricornutum* under nitrogen stress [28]. However, C-169 does not have the complete urea cycle as reported in diatom [28, 29]. Data here revealed that it employed alternative pathways to supply ornithine, and a different CPS, CPS II, to incorporate bicarbonate with glutamine to provide carbamoyl-phosphate for the ornithine pathway. The CPS-ornithine pathway together with GS/GOGAT cycle might represent an important pathway for anaplerotic carbon fixation with nitrogenous compounds, which were essential for amino acid and pyrimidine metabolism, as well as replenishing the TCA cycle.

The results reported here are important because they represent the first global transcriptomic analysis on the early stage of microalgae upon elevated CO_2_ and propose potential targets for future metabolic engineering. Metabolic pathway engineering has been actively explored to enhance microalgae-based biofuel production, which is mainly dependent on the knowledge of algal lipid accumulation [44]. Here, downregulation of lipid hydrolysis revealed by RNA sequencing and upregulation of FA synthesis indicated by qRT-PCR at the later stage, suggested genes directly involved in lipid biosynthesis and catabolism could be the target of metabolic pathway engineering. Similar approaches have been applied to different microalgae [44–46]. However, metabolic pathway engineering is calling for innovative and integrated strategies, and our results are enlightening to propose new gene targets for metabolic engineering. CO_2_ supplementation resulted in overexpression and enhanced activities of PEP carboxylase and pyruvate carboxylase, which could capture more carbon and reinforce TCA cycle. As a pivotal enzyme in central metabolism, downregulation of PEP carboxylase indicated that it controlled carbon flux distribution and decided the ratio of major biomass constituents [47]. Thus, PEP carboxylase and pyruvate carboxylase might be important candidates for metabolic engineering efforts to promote biomass production and synthesize desired bio-products. More than dozen V-ATPase subunits were markedly induced upon elevated CO_2_, which was postulated as an adaptive mechanism to maintain intracellular pH homeostasis. V-ATPases might be the potential targets to increase the CO_2_ tolerance in lipid-producing microalgae. Though the transcriptomic data here provide hints for metabolic engineering, it is obvious that the transcriptomic changes do not necessarily lead to changes in protein biosynthesis and enzyme activity, which might be due to post-transcriptional and post-translational regulation. Thus, these potential targets should be verified case by case in the future.

## Conclusion

In the present study, 2 and 5 % CO_2_ supplementation increased growth rate and lipid accumulation in autotrophically cultured *C. subellipsoidea* C-169. Overall biomass productivity of 222 mg L^−1^ day^−1^ and FA content as 48.5 % dry cell weight were found in 2 % CO_2_, suggesting C-169 as a great candidate for lipid production via CO_2_ supplementation. Transcriptomic profile comparison between 2 and 0.04 % CO_2_ unveiled the global regulation underlying rapid growth and lipid accumulation. C-169 enhanced gene expression in the Calvin cycle, and upregulated gene expression of PEP carboxylase, pyruvate carboxylase and CPS II to mobilize anaplerotic carbon assimilation pathways upon elevated CO_2_. Upregulation of ferredoxin and FNR implied that plentiful energy captured through photosynthesis might be converted and transferred through ferredoxin to sustain rapid growth and lipid accumulation. Upregulation of glycolysis, TCA cycle and oxidative phosphorylation gene expression implied them to provide abundant intermediates and metabolic energy for anabolism. Coordinated upregulation of nitrogen acquisition and assimilation genes, together with activation of CPS II and ornithine pathway genes might help C-169 to maintain C/N balance upon elevated CO_2_. Lipid accumulation was due to the significantly downregulated lipid degradation genes, as well as the upregulation of fatty acid synthesis genes at the later stage. Data here for the first time bring significant insights into the regulatory profile of metabolism and acclimation to elevated CO_2_ in C-169, which provide important information for future metabolic engineering to improve lipid production, and might eventually contribute to the development of sustainable microalgae-based biofuels.

## Methods

### Algal strain and culture conditions

*Coccomyxa subellipsoidea* C-169 was obtained from the Microbial Culture Collection of National Institute for Environmental Studies in Japan, under strain number NIES 2166. C-169 was incubated in 250-mL Erlenmeyer flasks containing 100 mL Bold’s Basal Medium (BBM) with continuous illumination provided by fluorescent light of ~60 μmol m^−2^ s^−1^ at 25 °C on an orbital shaker (130 rpm). The pre-culture was carried out at ambient level of CO_2_ (0.04 %, v/v) to reach logarithmic phase (OD_680_ = 0.8). The pre-cultured cells were subsequently transferred to fresh media with initial cell density of 2 × 10^6^ ml^−1^ and incubated with 0.04, 2 and 5 % CO_2_ (v/v) for 12 days. The pH of the medium was monitored using a pH meter (Mettler Toledo, Switzerland) and is provided in Additional file 2: Figure S2. Cells were sampled with 2-day interval followed by rinsing and centrifugation. Cell growth was monitored by counting cells with a hemocytometer. Dry cell weight was determined by weighing the cells pellet after lyophilization by a freeze drier (Modul YOD-230, Thermo-Fisher, USA).

### Analysis by flow cytometry and Confocal laser scan microscopy

Collected cells were resuspended at a density of ~5 × 10^6^ cells mL^−1^. Neutral lipids were quantified by Nile Red staining [48]. Aliquots of Nile Red (Sigma-Aldrich) in dimethyl sulfoxide (DMSO) were directly added to the suspension, having a final dye concentration of 2 μg mL^−1^ in 10 % DMSO (v/v). After incubation in dark for 3 min and filtration through a 45-μm membrane filter, all samples were analyzed using BD accuri C6 flow cytometer (BD Biosciences) equipped with 488-nm solid-state blue laser. The acquisition settings were 10^4^ events with medium flow rate (35 μL min^−1^, 16 μm core size). All settings were preliminary optimized. Fluorescence of Nile Red stained cells, chlorophyll auto-fluorescence were determined via FL2 (585/40 nm), FL3 (670 LP), respectively.

Fluorescence images of stained cells were captured by a Confocal Laser Scanning Microscope (CLSM, TCS SP5; Leica Microsystems CMS GmbH, Germany) under HCX PL APO CS 100× /1.4 oil-immersion objective with confocal pinhole set at Airy 1 and 5× zoom factor for improved resolution with eight bits. A blue excitation light was used through a band-pass filter (460–490 nm) and emission wavelengths were imaged through a long-pass filter (560–590 nm). Laser transmission and scan settings were constant in all scans.

### Total carbon content, carbon fixation rate and fatty acid profiling

C-169 cells from 0.04 % CO_2_ and 2 % CO_2_ were collected on the 4th, 8th and 12th day and lyophilized into cell pellets. Total carbon content (C_C_, % dry cell weight) was analyzed by an element analyzer (EuroEA3000, EuroVector S.p.A., Italy). CO_2_ fixation rate (${\text{R}}_{{{\text{CO}}_{{\text{2}}} }}$, g L^−1^ day^−1^) was determined as previously described [49]. It was calculated using the following equation: ${\text{R}}_{{{\text{CO}}_{{\text{2}}} }}$ = C_C_P(${\text{M}}_{{{\text{CO}}_{{\text{2}}} }}$/Mc), where P is the biomass productivity (g L^−1^ day^−1^), M_C_ is the molecular weight of carbon, and ${\text{M}}_{{{\text{CO}}_{{\text{2}}} }}$ is the molecular weight of CO_2_.

Fatty acid profiling was performed on the lyophilized cell pellets (Modul YOD-230, Thermo-Fisher, USA) via gas chromatography mass spectrometry (Agilent 6890 gas chromatography coupled with Agilent 5975 mass selective detector, Agilent Technologies, Santa Clara, CA, USA). Nonadecanoic acid (C19:0, Sigma-Aldrich, St. Louis, MO, USA) was added as internal standard to quantify FA content. Fatty acid methyl esters (FAMEs) were prepared and analyzed according to the protocol as previously described [50]. The degree of lipid unsaturation (DLU) was calculated according to previously described [51]:

$${\text{DLU}}({\triangledown }/{\text{mole}}) = \left[ {1.0 \, \times \, \left( {\% {\text{ monoene}}} \right) + 2.0 \, \times \, \left( {\% {\text{ diene}}} \right) + 3.0 \, \times \, \left( {\% {\text{ triene}}} \right)} \right]/100.$$

### RNA extraction, library construction and sequencing

Total RNA was extracted from AG and CG cells using TRIzol (Invitrogen, Carlsbad, CA, USA) and incubated with DNase I (Takara, Dalian, China) for 30 min at 37 °C. RNA quality and quantity were determined by a Nanodrop ND-1000 spectrophotometer (LabTech, Holliston, MA, USA) and Labon-chip analysis 2100 Bioanalyzer (Agilent Technologies, Santa Clara, CA, USA) (Additional file 2: Table S1). Approximately 10 μg of total RNA was subjected to poly(A) mRNA isolation with poly-T attached magnetic beads (Thermo-fisher). Following purification, mRNA was fragmented into small pieces using divalent cations under elevated temperature. Then the randomly cleaved mRNA fragments were constructed into cDNA library in accordance with the protocol for the Illumina RNA ligation-based method (Illumina, San Diego, USA). In brief, the fragmented RNA was dephosphorylated at the 3′ end by the phosphatase and phosphorylated at the 5′ end by the PNK. RNA was purified with the RNeasyMinElute Kit (Qiagen) and ligated with a pre-adenylated 3′ adapter, which enables the subsequent ligation of the 5′ adapter. Based on the adapter sequence, reverse transcription followed by PCR was used to create cDNA constructs. The average insert size for the paired-end libraries was 300 bp (±50 bp). The single end sequencing was then performed on Illumina Hiseq 2000.

### Sequencing data analysis

The raw data containing adaptor sequences, reads with low-quality sequences and unknown nucleotides N were filtered to obtain clean reads with 36 nt in length. Statistic analysis of data was provided in Additional file 2: Table S2. Clean reads were mapped to the transcript sequences of C-169 available on Phytozome V10 (http://www.genome.jgi.doe.gov/pages/dynamicOrganismDownload.jsf?organism=PhytozomeV10#) by Bowtie software [52], only 1 bp mismatch was allowed. For monitoring the mapping events on both strands, both the sense and the complementary antisense sequences were included in the data collection. The number of perfect clean reads corresponding to each gene was calculated and normalized to the number of Reads Per Kilobase of exon model per Million mapped reads (RPKM). Based on the expression levels, the significant DEGs (differentially expressed gene) between CG and AG were identified with |log_2_ fold change| > 1 and FDR < 0.001 unless otherwise noted. Functional classification of DEGs was conducted according to annotation in gene ontology (GO) and the pathway analysis was carried out according to KEGG. Heatmap clustering on top 100 DEGs of most significance was constructed and provided in Additional file 2: Figure S3.

### Quantitative RT-PCR

Quantitative RT-PCR was performed on ABI 7500 (Applied Biosystems, Foster City, CA, USA) using two-step kits (TaKaRa Biotech Co., Dalian, China). The gene coding ribosomal protein L5 (54775, RibL5) was used as an internal control according to the references [8, 53, 54] and the analysis of our transcriptomic data. For single-strand cDNA synthesis, the PrimeScript RT regent Kit with genomic DNA Eraser (TaKaRa) was used to perform the reverse transcription reaction according to the user’s manual. Genomic DNA removal was performed to purify the RNA extracts. Quantitative RT-PCR was performed with the SYBR Premix Ex Taq II kit (TaKaRa), based on cDNA template and 16 pairs of specific primers (Additional file 2: Table S3). Sequences of targeted genes were obtained form KEGG database and primers were designed using the Primer Premier 5.0 software. Primer alignments to secondary structures (predicted by mfold: http://www.unafold.rna.albany.edu/?q=mfold) of pairing sites and non-specific priming (validated by Primer-BLAST: http://www.ncbi.nlm.nih.gov/tools/primer-blast/) were avoided. Amplification program was 95 °C 30 s; 40 cycles at 95 °C for 5 s and 60 °C for 34 s followed by disassociation stage as instructed by user’s manual. Samples were performed in triplicate. The relative amount of gene transcripts was normalized to that of reference gene RibL5 in each sample. Expression fold change (FC) was calculated as:

FC_gene of x_ = 2^(Ct_AG_ – Ct_CG_) of gene x/2^(Ct_AG_ – Ct_CG_) of RibL5.

### Phosphoenolpyruvate carboxylase and pyruvate carboxylase activity assay

The enzyme activity assay was mainly as previously described with some modifications [55]. To analyze the enzyme activities, cell extracts from the 4th day were prepared by washing the cell pellets with TE buffer (10 mM Tris–HCl 1 mM EDTA, pH = 8.0) and broken with 0.1-mm-dia. silica beads in a mini-bead beater-1 (Biospec). Cell debris was removed by centrifugation at 12,000 rpm for 10 min at 4 °C. The supernatant was further centrifuged at 12,000 rpm at 4 °C for 20 min, and the resulting supernatant was used for the assay of enzyme activity. The phosphoenolpyruvate carboxylase (PEPCase) activity was determined by monitoring the decrease in absorbance of NADH of 340 nm using malate dehydrogenase as a coupling enzyme. The 1 ml reaction mixture for PEPCase analysis consisted of 50 mM HEPES (pH 7.3), 5 mM PEP, 10 mM MgCl_2_, 5 mM NaHCO_3_, 5U of malate dehydrogenase, 0.2 mM NADH, and 25 μl of cell extract. One unit of relative PEPCase activity was defined as 1 μM NADH being oxidized min^−1^ at 30 °C. The pyruvate carboxylase activity was similar to the method used above except using 5 mM pyruvate instead of PEP as substrate.

## Additional files

10.1186/s13068-016-0571-5 Transcriptomic data and DEGs enriched pathways or gene families.
10.1186/s13068-016-0571-5 Additional Tables and Figures.

## Abbreviations

DGE: digital gene expression
TAG: triacylglycerol
CLSM: confocal laser scanning microscope
FA: fatty acid
FAME: fatty acid methyl ester
DEGs: differentially expressed genes
AG: biological replicates from 0.04 % CO_2_ group
CG: biological replicates from 2 % CO_2_ group
RPKM: reads per kilobase exon model per million mapped reads
FC: fold change (CG to AG)
FDR: false discovery rate
GO: Gene Ontology
KEGG: Kyoto encyclopedia of genes and genomes
G6PD: glucose-6-phosphate dehydrogenase
6PGD: 6-phosphogluconate dehydrogenase
PGK: phosphoglycerate kinase
GAPDH: glyceraldehyde 3-phosphate dehydrogenase
TPI: triosephosphate isomerase
ALDO: fructose-bisphosphate aldolase
FNR: ferredoxin–NADP^+^ reductase
SP: starch phosphorylase
PFK: 6-phosphofructokinase
GAPN: glyceraldehyde-3-phosphate dehydrogenase (NADP^+^)
PK: pyruvate kinase
FBP: fructose-1,6-bisphosphatase
PEPCK: phosphoenolpyruvate carboxykinase
CS: citrate synthase
IDH3: isocitrate dehydrogenase 3
OGDC: 2-oxoglutarate dehydrogenase complex
NR: nitrate reductase
NIR: nitrite reductase
GS: glutamine synthetase
GOGAT: glutamine 2-oxoglutarate aminotransferase
GLDH: glutamate dehydrogenase
CPS: carbamoyl-phosphate synthase
OTC: ornithine carbamoyltransferase
ASS: argininosuccinate synthase
ASL: argininosuccinate lyase
ACS: acyl-CoA synthetase
ACOX: acyl-CoA oxidase
MFP-2: enoyl-CoA hydratase/3-hydroxyacyl-CoA dehydrogenase
ACAT: acetyl-CoA acyltransferase
