# Supplementary material for: Transcriptome analysis reveals global regulation in response to CO2 supplementation in oleaginous microalga Coccomyxa subellipsoidea C-169
Source: Biotechnol Biofuels. 2016 Jul 22;9:151. doi: 10.1186/s13068-016-0571-5 (PMC4957332; doi:10.1186/s13068-016-0571-5)
Supplement: Supplementary file 2 — 10.1186/s13068-016-0571-5 Additional Tables and Figures. [file 13068_2016_571_MOESM2_ESM.docx]

**Table S1** Quality control assessment on extracted RNA from the replicates of AG and CG

| Sample | Conc. (µg/µL) | UV 260/280 | UV 260/230 | Amount (µg) | RIN |
| --- | --- | --- | --- | --- | --- |
| AG_1 | 0.47 | 2.15 | 2.47 | 21.16 | 8.0 |
| AG_2 | 0.33 | 2.10 | 2.48 | 14.85 | 7.2 |
| AG_3 | 0.44 | 2.15 | 2.48 | 19.61 | 7.4 |
| CG_1 | 0.41 | 2.05 | 2.39 | 18.41 | 8.1 |
| CG_2 | 0.40 | 2.07 | 2.41 | 18.08 | 8.8 |
| CG_3 | 1.01 | 2.14 | 2.47 | 45.65 | 7.4 |

RIN (RNA Integrity Number) is used to indicate the RNA quality.

**Table S2** DGE library profile of replicates from AG and CG

| Sample | Total reads | Unique reads | Aligned reads | % of aligned reads | No, unique genes hit |
| --- | --- | --- | --- | --- | --- |
| AG_1 | 8544926 | 5767453 | 3965553 | 46.42% | 9177 |
| AG_2 | 8664050 | 6006599 | 3825993 | 44.17% | 9142 |
| AG_3 | 9999804 | 6583079 | 4969639 | 49.70% | 9190 |
| CG_1 | 5854690 | 4485058 | 2674507 | 45.69% | 9067 |
| CG_2 | 5759178 | 4410504 | 2724210 | 47.31% | 9100 |
| CG_3 | 7926569 | 5774762 | 3740989 | 47.20% | 9132 |

**Table S3** Primer sequences used in the quantitative RT-PCR analysis

| Primers | Sequence (5'–3′) | Product size (bp) |
| --- | --- | --- |
|  |  |  |
| 54775 60S ribosomal protein L5 | CACGTCTGAGGCTTACTACCC  TCTCCAGCAACAGTGGCATA | 121 |
| 31164 ferredoxin | TACCCCGCTGTCTTCTGTTG  GACCTTGTCTGCTTCATC | 142 |
| 4465 FATA | TCTTTGCTGTGACCAGAATGC  GATGATGATCCAGTTGCGG | 125 |
| 65159 ACCase | CTTGCTTTTGACGAGCTGACT  TTGCTGGTAGACGACTGTTGG | 147 |
| 49000 fatty acid synthase I | AAGGAGTATGGGATTGTGCC  TGTGGTAGGCGATGAGGATG | 115 |
| 54810 3-oxoacyl-ACP synthase II | TGCCTCGGTCTTTGGTAATG  CCCCTCGTTGTCAAAGTTCTC | 145 |
| 25960 V-type H+-transporting ATPase subunit A | GCCTTCTGACCACCATT  GCAAACTCATAGCCCTTCTTCC | 130 |
| 26440 pyruvate carboxylase | CGGGACCGTTGAGTTTATGG  TGCAATCCTGATCTGAGCCT | 136 |
| 26453 GAPDH | GACAAGGCCAACTCACACATAAAG  CGTCGTACTTCTCCTCGTTCA | 109 |
| 27575 ACCase | TGTCCCCACAGACTGCTCAT  CAGGGTTGGTCTTCTTGCTC | 147 |
| 39093 G6PD | ACCTACAAGCAGCGATACAACA  CATGCAGCGTCCAACTCA | 116 |
| 52173 PEPCase | GTTTGGGGAGAAGGAGGTGT  ATTTCGTTGAGGACGGAGC | 120 |
| 54043 acetyl-CoA acyltransferase | GGAGAATGTGGCGGAGAAGT  GGCACGATCTCGTCCTTGA | 120 |
| 54860 CPS II | TCACGGGCTACCAGGAGATT  CCACCCAGATGGCACTTTTC | 130 |
| 65708 RuBisCO | GGAGCCCGTTGACAACAAGT  CGTTCGAGAACTCCAGAGCA | 128 |
| 47508 fatty acid synthase, animal type | GGCATTCTTCTCAGTCCACG  CACATACACTCCCACCTTCTCG | 145 |

**
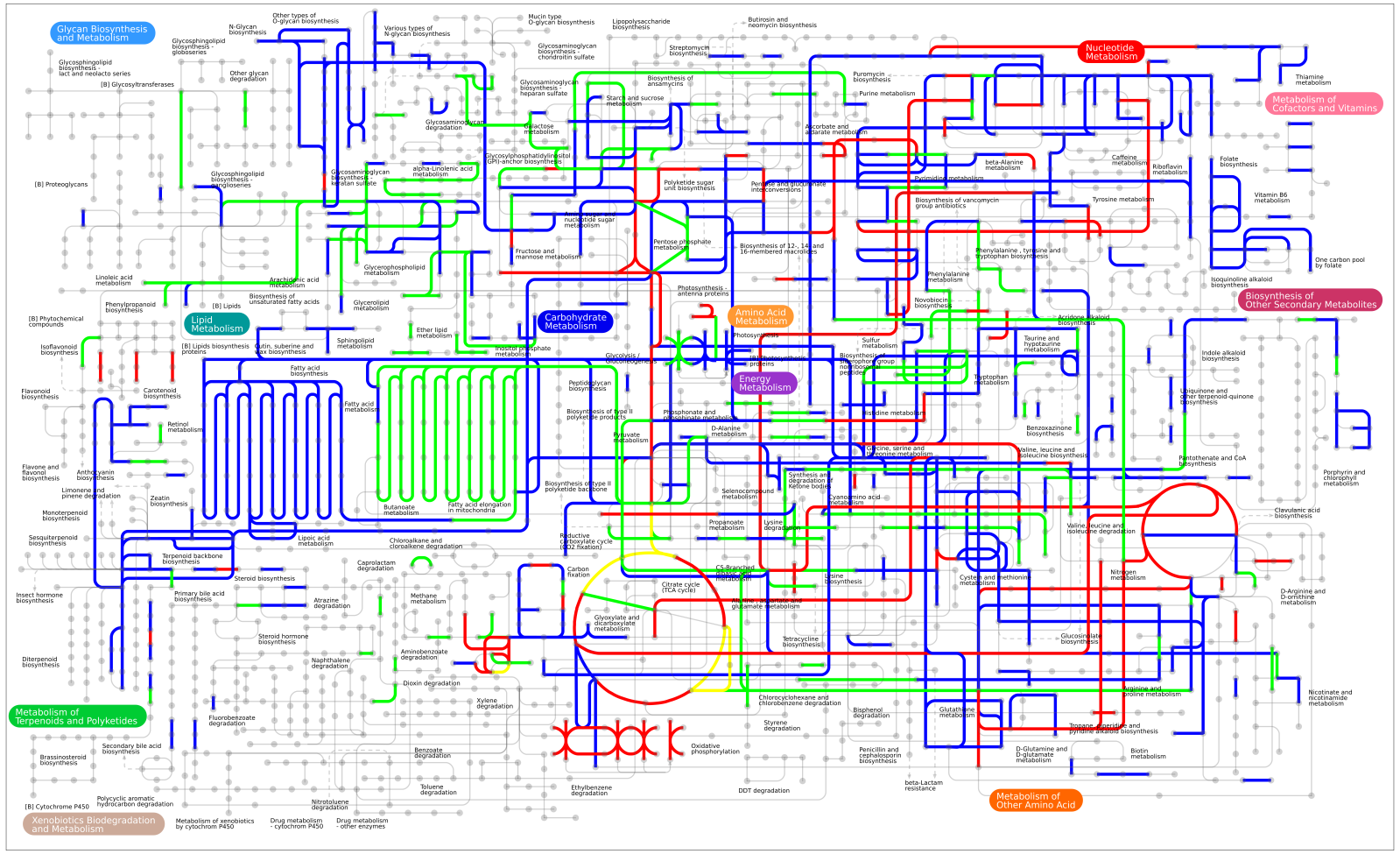
**

**Figure S1 Global metabolic pathways of differentially expressed genes in response to CO_2_ supplementation**. The gray lines depict pathways not present or not yet assigned in C-169 and the blue lines represent pathways that showed no significant changes in transcription. Pathways enriched in genes that are up-regulated or down-regulated are labeled in red and green, respectively. The yellow lines represent pathways in which both up-regulated and down-regulated genes are involved. Significance is defined as |log_2_ FC| >1 and FDR < 0.001.


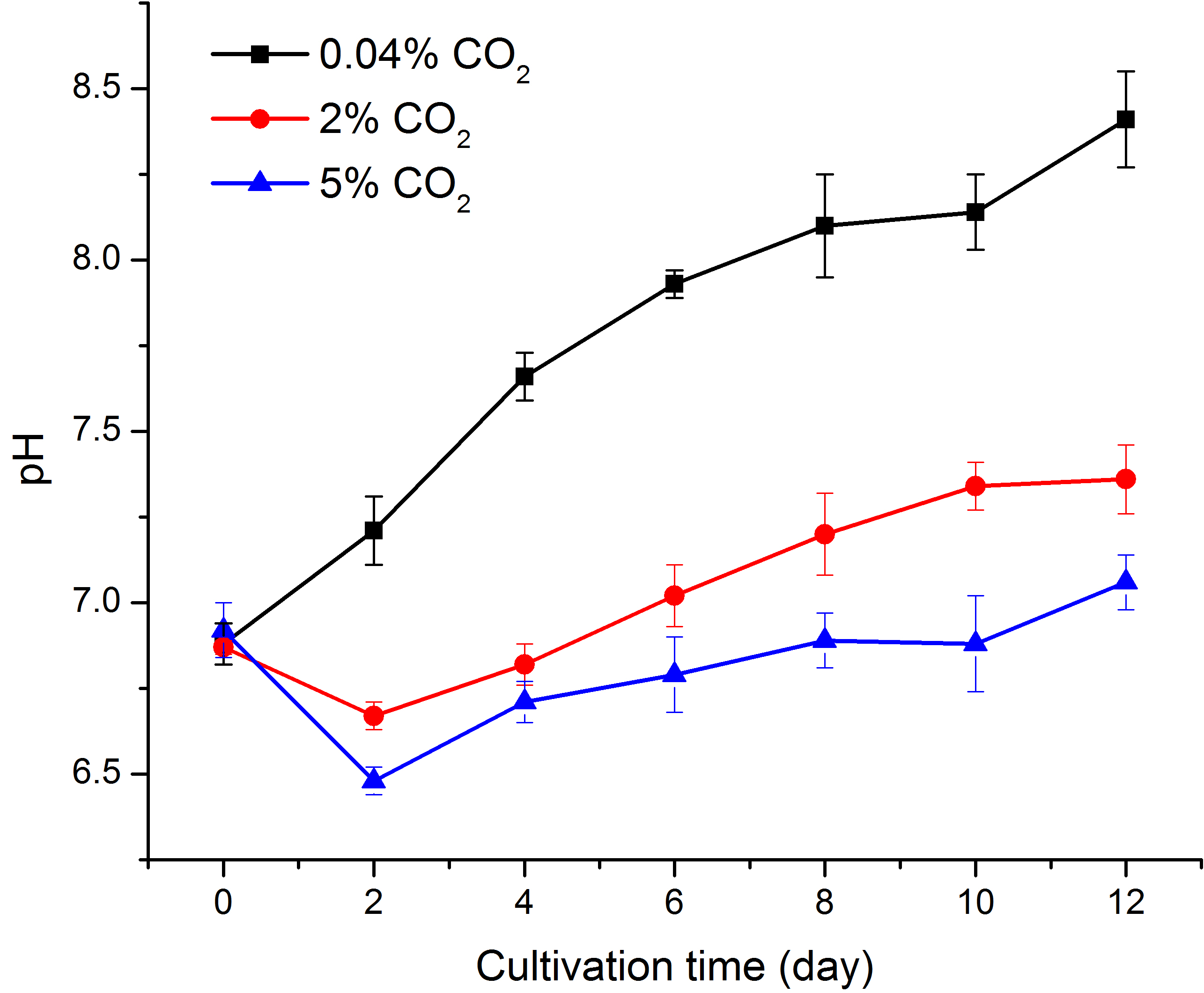


**Figure S2 The pH changes in the media of C-169 under different CO_2_ concentrations.**

**Figure S3 Heatmap clustering on top 100 genes of most significance (indicated by FDR, false discovery rate) from AG and CG.** Heatmap was generated via pheatmap package in R.
